# Comparison of Time Course Detection of Human Male DNA from Blood Stains on Various Objects on Surface in a Natural Environment and in a Laboratory Using Loop-Mediated Isothermal Amplification (LAMP)

**DOI:** 10.1155/2021/4811608

**Published:** 2021-11-25

**Authors:** Ratchanok Kumsiri, Panan Kanchanaphum

**Affiliations:** ^1^Pathobiology Unit, Faculty of Science, Rangsit University, Pathumthani, Thailand; ^2^Biochemistry Unit, Faculty of Science, Rangsit University, Pathumthani, Thailand

## Abstract

In forensic study, the biological evidence can easily degrade, especially DNA. Degraded and environmentally challenged samples can produce numerous problems in forensic DNA analysis including loss of band product. Loop-mediated isothermal amplification or LAMP is one of the DNA analysis techniques used in forensic study. This study explores the limitations of the efficiency of the LAMP technique on abandoned DNA. For the DNA template, 8 male and 2 female blood-stained samples were taken from the surfaces, namely, brick, cloth, and tile from inside, and buried outside the laboratory. The LAMP reaction was used to amplify the SRY gene for detecting male DNA. All the blood-stained samples were stored for 1, 7, 15, 30, and 45 day (s). The LAMP product from the blood-stained samples on all the surfaces that were kept in a laboratory was detected using the gel electrophoresis technique from day 1 until day 45. However, the LAMP product on day 30 and 45 was smear and dim. The LAMP product from the blood-stained samples buried outside the laboratory was observed using the gel electrophoresis technique within day 30 (smear and dim). To increase the efficiency of detection, the qLAMP technique detected product on all the male samples from all the surfaces buried outside the laboratory for 45 days. The results indicate that this LAMP condition was possible detecting male DNA and the environmental factors are the main influence on the sensitivity of the LAMP technique. In addition, the qLAMP technique can increase the capacity and sensitivity of the detection.

## 1. Introduction

Currently, loop-mediated isothermal amplification (LAMP) is used in forensic studies such as in human sex determination [1–3] and human identification [4]. This technique is *in vitro* DNA amplification that uses at least 2 pairs of specific primer, at a single temperature combined with Bst DNA polymerase for 45–60 minutes. At least two sets of primer (inner primer and outer primer sets) used in LAMP are specific at six different regions located within the target sequence, and primary DNA amplification begins by the inner primer set. The characteristic intermediary DNA structure formed by LAMP, called a stem-loop DNA fragment, is generated, and large amounts of DNA products were produced by an autocycle reaction [5]. LAMP has many advantages over conventional methods such as the polymerase chain reaction (PCR). It is less complicated and less time consuming, and there are many amplification product detection methods such as gel electrophoresis, the naked eye of magnesium pyrophosphate precipitation, and the lateral flow dipstick (LFD) [5].

Kanchanaphum [2] showed the efficiency of human male DNA detection from blood-stained samples on various surfaces such as cloth, wood plank, clay, and tile using the LAMP technique. This study was performed under laboratory conditions (a constant temperature at 25°C and 50% relative humidity (RH)). However, in real life, evidence and samples are usually taken from environments that are weathered or contaminated. Biological evidence can easily degrade, especially DNA. Degraded and environmentally challenged samples can produce numerous problems in forensic DNA analysis including loss of band product. However, DNA degradation is not the only issue encountered when analyzing challenging samples. Many such samples contain substances that are coextracted with the DNA and inhibit the PCR reaction. The limitations of LAMP efficiency under poor conditions have not been reported.

The major site of an oxidative attack on the DNA bases is the C=C double bond of pyrimidines and purines, leading to ring fragmentation and base modifications. Many of these oxidized base products will block replication, negatively impacting amplification with the standard Taq-DNA polymerases used in PCR [6].

Degraded and environmentally challenged samples can produce numerous problems in forensic DNA typing including loss of signal, peak imbalance, and allele dropout. However, DNA degradation is not the only issue encountered when analyzing challenging samples. Many such samples contain substances that are coextracted with the DNA and inhibit the PCR reaction. Therefore, this study determines the limitation of the efficiency of the LAMP technique on abandoned DNA.

## 2. Materials and Methods

### 2.1. DNA Template Preparation

Before starting the experiment, the Ethic Review Board of Rangsit University has granted ethical approval for this study (COA. No. RSUERB2019-072). All DNA samples from all stained blood were prepared by smearing 2-3 drops of blood sample or about 300 *μ*l (8 male and 2 female) to surface material (brick, cloth, and tile), letting them to dry and store the sample on the table in the laboratory room as the control room (CR) which controls temperature at 25°C and moisture level at 80%. Another set of samples which is the same as the first set was placed outside laboratory room (OR) which do not control temperature and moisture content and buried about 15 cm under the soil located outside the laboratory room. The DNA template preparation step was followed by Kanchanaphum et al. [1]. We started the experiment by smearing the blood samples to each surface both inside and outside laboratory room at the same day. The cotton bud was submerged into 200 *μ*l of distilled water. The distilled-water-soaked cotton bud was swabbed on the surface material for collecting blood stained. Then, this cotton bud was submerged into a tube which contained 500 *μ*l of distilled water. After that, this solution was used for DNA extraction by using the GF-1 Blood DNA extraction kit (Vivantis, Selangor Darul Ehsan, Malaysia).

For time course detection, the eight males' and two females' blood-stained samples were spotted on 3 types of surface material and stored for 1, 7, 15, 30, and 45 days in a control room (CR) and outside room (OR). Then, the blood-stained samples were used for DNA extraction by the method mentioned above. Afterward, these DNA solutions were used as template for LAMP amplification.

### 2.2. LAMP and qLAMP Reaction and Analysis

The SRY primers used in this study were design based on the human SRY gene (GenBank accession No. JQ811934) and as previously described in [2]. All LAMP reactions were carried out as described previously in detail. All reactions were carried out in 25 *μ*l of 1x *Bst* DNA polymerase buffer containing 5 mM MgSO_4_ (MERK, Kenilworth, USA), 400 mM betaine (MERK, Kenilworth, USA), 1.2 mM dNTPs (Vivantis, Selangor Darul Ehsan, Malaysia), 0.8 *μ*M F3 and B3 primers, 2 *μ*M FIP and BIP primers, and 8 U *Bst* DNA polymerase (New England Biolabs, Ipswich, USA), and 5 *μ*l of DNA template. Reactions were incubated at 65°C for 45 min and followed by enzyme inactivation at 80°C for 5 min. The conventional LAMP reactions were performed in a MiniAmp Plus Thermal Cycle (Applied Biosystems by Thermo Fisher Scientific, Waltham, Massachusetts, USA). After amplification, the LAMP products were analyzed by loading 10 *μ*l of LAMP product on 1.5% agarose gel. After gel electrophoresis, the LAMP product was analyzed by Gel Doc^TM^ XR + with Image Lab^TM^ Software (BIO-RAD, Hercules, USA). The qLAMP amplification was performed in the CFX Connect Real-Time system (BIO-RAD, Hercules, USA) by adding 0.5 *μ*L of SYBR green I dye (Invitrogen, Waltham, USA) to the normal LAMP reaction (only the samples which were stored outside the laboratory room at day 45).

## 3. Results

**Figure 1:**
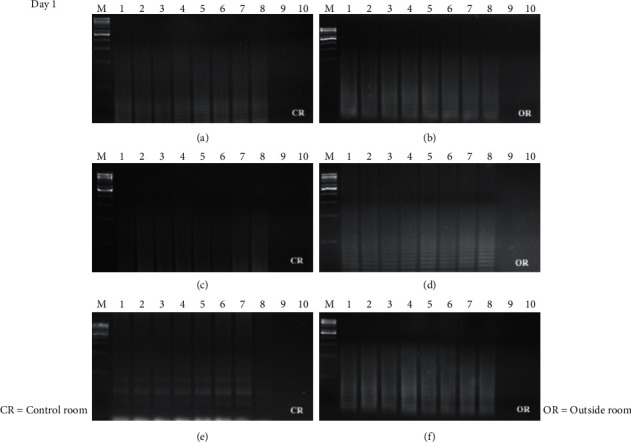
Time course detection of DNA sample stained on different surfaces in day 1. (a), (b) brick, (c), (d) cloth, and (e), (f) tile. *M* = 1 kB DNA ladder, lane 1–9 = male sample 1–8, and lane 9, 10 = female sample 1-2.

**Figure 2:**
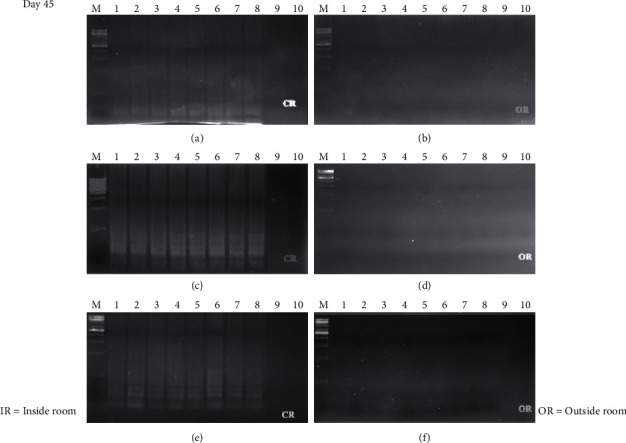
Time course detection of DNA sample stained on different surfaces in day 45. (a), (b) brick, (c), (d) cloth, and (e), (f) tile. *M* = 1 kB DNA ladder, lane 1–9 = male sample 1–8, and lane 9, 10 = female sample 1-2.

**Figure 3:**
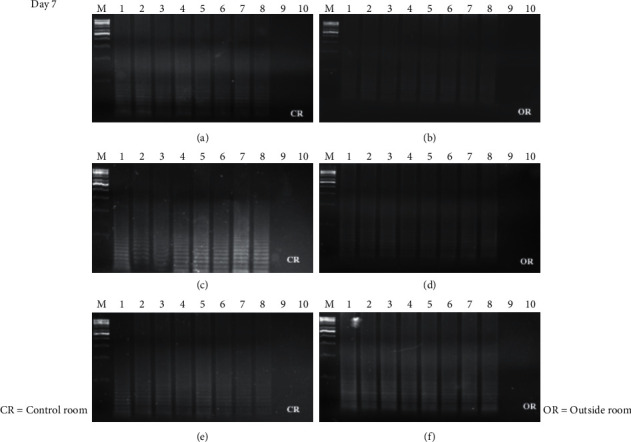
Time course detection of DNA sample stained on different surfaces in day 7. (a), (b) brick, (c), (d) cloth, and (e), (f) tile. *M* = 1 kB DNA ladder, lane 1–9 = male sample 1–8, and lane 9, 10 = female sample 1-2.

**Figure 4:**
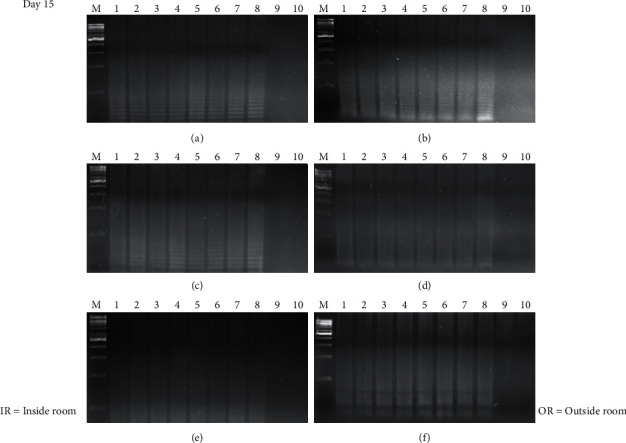
Time course detection of DNA sample stained on different surfaces in day 15. (a), (b) brick, (c), (d) cloth, and (e), (f) tile. *M* = 1 kB DNA ladder, lane 1–9 = male sample 1–8, and lane 9, 10 = female sample 1-2.

**Figure 5:**
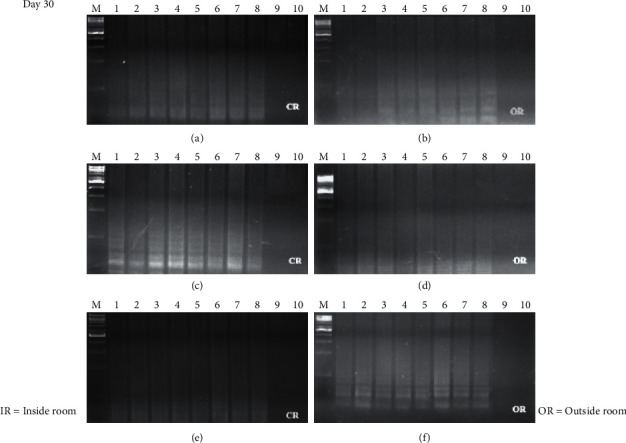
Time course detection of DNA sample stained on different surfaces in day 30. (a), (b) brick, (c), (d) cloth, and (e), (f) tile. *M* = 1 kB DNA ladder, lane 1–9 = male sample 1–8, and lane 9, 10 = female sample 1-2.

**Figure 6:**
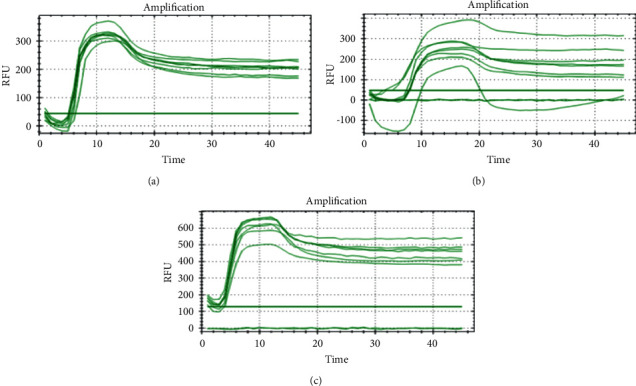
Time course detection of DNA sample stained on different surfaces in day 45 by qLAMP. (a) = brick, (b) = cloth, and (c) tile.

The time course detection comparison for the three types of surface material is shown in Figures 1(a), 1(c), and 1(e)to 2(a), 2(c), and 2(e) from the control room (CR) and Figures 1(b), 1(d), and 1(f)to 2(b), 2(d), and 2(f) from buried outside (OR). On day 1 and day 7, the LAMP product signals were strong for all the samples, as shown in Figures 1 and 3. On day 15, the LAMP product from all the samples in the laboratory room except for the tile surface was obvious, as shown in Figures 4(a) and 4(c). However, the product on the tile surface became faded (control room) and did not have a ladder pattern band, as shown in Figure 4(e). All LAMP products kept outside became faded and appeared as a smeared band (Figures 4(b), 4(d), and 4(f)). On day 30, the LAMP product from the laboratory room was a smeared band, as shown in Figures 5(a), 5(c), and 5(e). All the LAMP product samples from outside were smeared and dim, especially on the surface of the brick where the LAMP product had faded (Figures 5(b), 5(d), and 5(f)). On day 45, the LAMP product on the surface of the brick had disappeared. Similarly, the LAMP product on the surface of the cloth and tile had faded, as shown in Figures 2(a), 2(c), and 2(e). On the other hand, on the samples buried outside, the product had vanished (Figures 2(b), 2(d), and 2(f)). Therefore, all the DNA samples from outside of day 45 were analyzed using the qLAMP technique. The qLAMP results are shown in Figure 6. The comparison of LAMP product detection using gel electrophoresis in location, surface, and time is shown in Table 1. In lane 9 and 10 of all experiments were female samples, and LAMP products did not appear.

## 4. Discussion

In real crime investigations, the evidence from crime scenes can be damaged by environmental factors such as temperature, humidity, and chemicals. This study demonstrates the limitations of the LAMP technique at distinguishing human male DNA samples kept in a natural environment. The LAMP technique still detected DNA on male samples on day 30 on all the surfaces kept in the laboratory and outside the laboratory using the technique adapted by Kanchanaphum [2] which detected male DNA on samples using LAMP on day 30. The results demonstrated the limitations of the LAMP technique on all surfaces in the laboratory on day 45. All LAMP products were observed by gel electrophoresis; however, the LAMP product was faded and had a ladder pattern. While outside the laboratory on day 45, none of the LAMP product was observed on all surfaces. However, when all DNA samples buried outside the laboratory were analyzed using qLAMP, the LAMP product was detected which agrees with the work of Kumsiri and Kanchanaphum [7] and Vichaibun and Kanchanaphum [8]. Their results indicated that the efficiency and sensitivity of qLAMP were better than those of conventional LAMP.

From this study, we used 2-3 drops of blood sample or about 300 *μ*l. Normally, the amount of DNA from whole blood per ml is about 260–1,474 ng/ml [9] or 1,700 leucocyte-eq/ml [10]. Therefore, the 300 *μ*l of blood sample which used in this experiment is equal to 78–441 ng of DNA or 510 leucocytes. It means that our LAMP condition can detect at least 260–1,474 ng of DNA or 170 leucocytes.

Several environmental factors affected DNA degradation such as temperature, chemicals, and humidity. The samples kept outside the laboratory were affected by sun exposure as the temperature was about 37–40.5°C. This temperature is not high enough to damage DNA according to Bauer et al. [11] who studied the effect of temperature and pH on the degradation of DNA and noted that the nicking of DNA was observed at more than 65°C. Therefore, in this case, the temperature did not affect the stability of DNA on the surface. No LAMP products visualized by gel electrophoresis were detected in all samples from day 45 outside the laboratory, as shown in Figures 5(b), 5(d), and 5(f).

Another interesting factor is the effect of chemicals such as humic substances. Humic substances are important soil components [12] and are encountered in samples that have been buried. The condensation of biomass of microorganisms, plants, and animals is usually also found in the soil. The DNA from the buried samples outside the laboratory may have been contaminated with humic substances and biomass. The DNA contamination by humic substances has resulted in the DNA amplification process, endonuclease restriction reaction (18), and difficulties in DNA extraction [13, 14]. And some microorganisms found in the soil such as bacteria can digest DNA [15].

Besides humic substances, indigo is another interesting chemical that is found in fabric and cloth that acts as an inhibitor in the DNA extraction step [16]. The DNA amplification product could not be detected using real-time instruments due to interference by the dark blue color of the reaction mixture and the real-time results indicating a loss of efficiency that was possibly related to the quenching of the dye [16].

## 5. Conclusions

These results of this study pinpoint that a variety of environmental factors such as microorganisms, and some chemicals affect the stability of DNA. Consequently, the LAMP reaction efficiency was decreased. However, the period that the samples are kept in the environment is also an important factor. The LAMP reaction efficiency decreased as the period the samples were kept outside increased. Nonetheless, qLAMP can increase the detection performance of conventional LAMP.

## Figures and Tables

**Table 1:** Comparison of LAMP detection using gel electrophoresis in location, material surface, and day.

Location	Laboratory room			Buried and outside		
	Brick	Cloth	Tile	Brick	Cloth	Tile
Day 1	✓	✓	✓	✓	✓	✓
Day 7	✓	✓	✓	✓ (smear)	✓ (smear)	✓
Day 15	✓	✓	✓ (smear)	✓ (smear)	✓ (smear)	✓ (smear)
Day 30	✓ (smear)	✓ (smear)	✓ (smear)	✓ (smear/dim)	✓ (smear)	✓ (smear)
Day 45	✓ (smear/dim)	✓ (smear)	✓ (smear)	—	—	—

## Data Availability

The data used to support the findings of this study are available from the corresponding author upon request.
